# Increased physical activity promotes skin clearance, improves cardiovascular and psychological health, and increases functional capacity in patients with psoriasis

**DOI:** 10.1002/ski2.426

**Published:** 2024-08-09

**Authors:** Rory Sheppard, Weh K. Gan, Gladys L. Onambele‐Pearson, Helen S. Young

**Affiliations:** ^1^ Division of Musculoskeletal and Dermatological Sciences School of Biological Sciences The University of Manchester Manchester UK; ^2^ The Dermatology Centre Salford Royal Hospital Manchester Academic Health Science Centre The University of Manchester Manchester UK; ^3^ Department of Sport and Exercise Sciences Musculoskeletal Science and Sports Medicine Research Centre Manchester Metropolitan University Manchester UK

## Abstract

**Background:**

Patients with psoriasis are less physically active compared to age‐matched controls, due to psoriasis‐specific barriers, which significantly limits their ability to benefit from health‐promoting levels of physical activity (PA). In addition, long‐term health outcomes for people with psoriasis are poor and include depression, metabolic syndrome and cardiovascular disease (CVD); presenting a significant challenge to healthcare services.

**Objectives:**

We designed a PA intervention in partnership with patients with psoriasis hypothesising this may have therapeutic utility in the management of psoriasis.

**Methods:**

Participants with chronic plaque psoriasis were recruited to a single‐centre, 20‐week, prospective cohort study. A wrist‐worn accelerometer (GENEActiv Original; Activinsights Ltd) and a hip‐worn pedometer (Onwalk 900; Decathlon Group) were used objectively measure levels of PA. Our 10‐week PA intervention comprised twice weekly 60‐min walks within three different greenspaces in Greater Manchester, each led by a Sports and Exercise Scientist to deliver a pre‐specified volume/dose of activity. During weeks‐11–20 of the study, participants followed independent activities. Clinical evaluation, including assessment of psoriasis severity, cardiometabolic parameters, psychological wellbeing and functional capacity was made at baseline, week‐10 and ‐20.

**Results:**

Sixteen patients with psoriasis completed the study. We observed significantly reduced Psoriasis Area and Severity Index at week‐10 (*p* = 0.01) and ‐20 (*p* = 0.001) compared to baseline, with 50% of participants achieving PASI‐50 at week‐20. Dermatology Life Quality Index (DLQI) was significantly reduced at week‐20 (*p* = 0.04), compared to baseline. Significant reduction in blood pressure at week‐10 (systolic: −7.4 mmHg, *p* = 0.002; diastolic: −4.2 mmHg, *p* = 0.03) and ‐20 (systolic: −8.8 mmHg, *p* = 0.001; diastolic: 4.1 mmHg, *p* = 0.008) was observed and pulse wave velocity was significantly reduced by week‐20 (*p* = 0.02), suggesting improvement in cardiovascular health. Despite high prevalence of anxiety and depression at baseline, we documented a significant improvement in wellbeing and psychological health. Functional capacity was significantly enhanced following completion of the study.

**Conclusion:**

Increasing PA constitutes a promising therapeutic intervention in the management of psoriasis. Evaluation of our intervention in a clinical trial would help determine clinical utility and establish PA guidelines for patients with psoriasis.

## Graphical abstract

Increased physical activity promoted significant skin clearance in patients with psoriasis with 50% of our cohort achieving a PASI‐50 response at week‐20. We also observed a significant improvement in cardiometabolic disease status, psychological health, and functional capacity. In summary, facilitating increased physical activity in patients with psoriasis constitutes a promising therapeutic intervention in disease management and warrants further evaluation in a clinical trial.

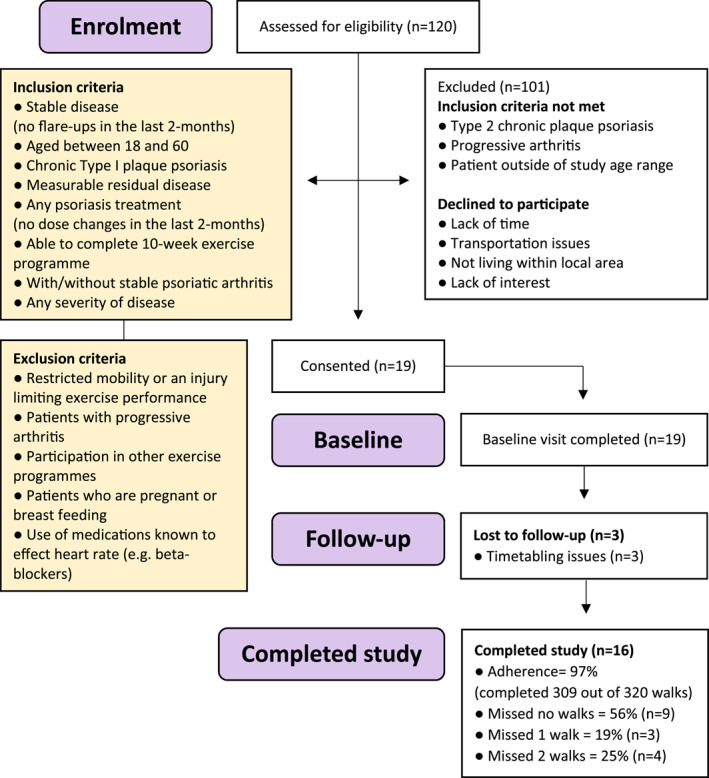

**What is already known about this topic**

- Patients with psoriasis face psoriasis‐specific barriers which limit participation with physical activities.
- Psoriasis is associated with significant psychosocial impairment and comorbidities such cardiovascular disease (CVD), and inflammatory arthritis.
- The anti‐inflammatory effect of increasing Physical activity (PA) may have utility in the management of psoriasis and psoriatic comorbidities.
- The impact of increasing PA on health‐related parameters has not been investigated in a psoriasis population.

**What does this study add?**

- Our evidence‐based programme was developed in partnership with patients with psoriasis to specifically remove barriers prohibiting engagement with physical activities for those with psoriasis.
- Increased PA promoted significant improvement in psoriasis with 50% of our cohort achieving a PASI‐50 response at week‐20. We also observed a significant improvement in cardiometabolic disease status, psychological health, and functional capacity.
- Evaluation of PA in a randomised controlled clinical trial would help determine clinical utility.

## 1 INTRODUCTION

We have previously reported that patients with psoriasis are less physically active than those who do not have psoriasis due disease‐specific barriers such as psoriasis severity, skin sensitivity, clothing choice, participation in social/leisure activities, and treatments.^1^, ^2^ Indeed, In comparison to data from The Health Survey for England^3^ significantly more patients with psoriasis (60% females with psoriasis; *p* = 0.003; and 45% males with psoriasis; *p* = 0.011) failed to exercise to the extent recommended by current guidelines.^1^, ^4^ This means patients with psoriasis are unable to benefit from the health‐promoting levels of physical activity advocated in current guidelines,^4^ and may augment the elevated risks of cardiovascular disease (CVD) and metabolic syndrome reported in this group.^1^

Despite a significant improvement in the development of effective psoriasis therapies,^5^ unfavourable outcomes remain common due to poor treatment tolerability, a lack/loss of treatment efficacy, and possible undertreatment of patients.^6^ Promisingly, increased PA ameliorates a pro‐inflammatory phenotype^7^ and strategies to increase PA in those with other immune‐mediated inflammatory diseases, such as inflammatory arthropathy, CVD, metabolic syndrome, and depression/anxiety have offered clinical benefits.^8^ Indeed, patients with psoriasis, working in collaboration with healthcare professionals, recently identified the role of lifestyle factors in the management of psoriatic disease as the most important research priority in both the Psoriasis^9^ and Psoriatic Arthritis Priority Setting Partnerships^10^; James Lind Alliance facilitated collaborations.

Therefore, having identified that patients, because of their disease, are less likely to engage in physical activities and are restricted in accessing leisure facilities we sought to address this by designing a PA intervention in partnership with those with lived‐experience as previously described.^11^ Hypothesising that increased PA may have therapeutic utility in the management of psoriasis, we then measured the clinical impact of our PA intervention in a proof‐of‐concept study, investigating health outcomes including psoriasis control; CVD/metabolic syndrome risk; wellbeing/psychosocial functioning and functional capacity.^11^

## 2 MATERIALS AND METHODS

### 2.1 Recruitment and study design

**FIGURE 1:**
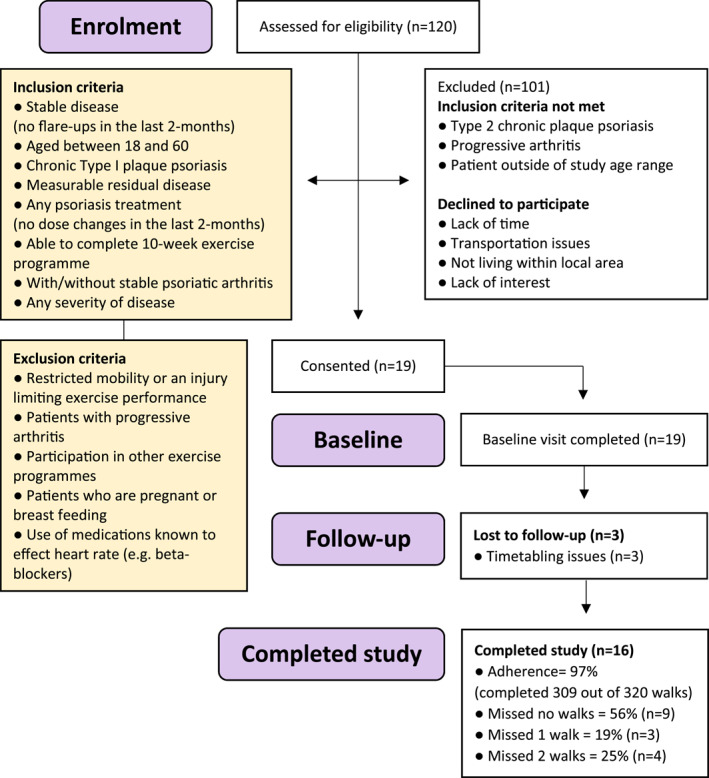
Participants with Type 1 chronic plaque psoriasis with or without psoriatic arthritis were recruited to a single‐centre, 20‐week, prospective cohort study, with a single experimental arm. Participants were excluded from the study if they had rapidly worsening inflammatory arthritis, or restriction in mobility/injury limiting capacity for physical activities. The final cohort (*n* = 16) comprised equal numbers of men and women.

Participants (*n* = 19) with Type 1 chronic plaque psoriasis with or without psoriatic arthritis (PsA) were recruited to a single‐centre, 20‐week, prospective cohort study, with a single experimental arm (Figure 1). The study was approved by the Local Research Ethics Committee (20/NW/0443) and was conducted in accordance with the Declaration of Helsinki principles following informed consent. Participants were excluded from the study if they had rapidly worsened inflammatory arthritis, or restriction in mobility/injury limiting capacity for physical activities (Figure 1). Following recruitment participants were provided with a wrist‐worn accelerometer (GENEActiv Original; Activinsights Ltd) and a hip‐worn pedometer (Onwalk 900; Decathlon Group, Villeneuve d’Ascq, France) to objectively measure levels of PA for one week prior to and during the study. Both devices have good reliability and have been validated in previous studies. Data collection took place between May 2021 and July 2023.

### 2.2 The physical activity intervention

Our 10‐week PA intervention, co‐designed with those having lived‐experience of psoriasis as previously described,^11^ comprised a suite of twice weekly 60‐min walks within three different greenspaces in Greater Manchester, each led by a Sports and Exercise Scientist.^11^ Each session was limited to a small‐group of participants (*n* = 2–4) and was structured to ensure delivery of a pre‐specified volume/dose of activity. This meant participants could incrementally progress from a relatively sedentary baseline to heart‐healthy levels of activity (as defined by the American Heart Asosciation totalling an energy expenditure of ≥500 MET‐minutes/week) within 4 weeks of programme participation. Heart‐healthy levels of PA were then sustained for the remainder of the intervention.^11^ Warm‐ups, pre‐activity stretching, and cool‐downs were also incorporated into each session. During weeks‐11‐20 of the study, participants followed independent activities and recorded these in PA diaries.

### 2.3 Clinical evaluation of functional capacity, psoriatic/cardiometabolic disease, and psychosocial wellbeing

Participants had a detailed clinical evaluation at baseline, after which they followed our 10‐week PA intervention. Further clinical evaluation was made after completion of the PA intervention (week‐10) and on completion of the study (week‐20). Throughout the study participants continued with their usual psoriasis treatment.

#### 2.3.1 Assessment of psoriasis and psoriatic arthritis

A clinical history was taken from each participant and assessment of psoriatic disease was documented using Psoriasis Area and Severity Index (PASI; 0–72),^12^ Dermatology Life Quality Index (DLQI; 0–30 points),^13^ physicians global assessment (PGA; 0–7 points), Psoriatic Arthritis Impact of Disease 12‐item questionnaire (PsAID‐12; 0–10),^14^ and Routine Assessment of Patient Index Data 3 (RAPID3; 0–30 points).^15^

#### 2.3.2 Cardiometabolic assessment

Anthropometric measurements including height, weight, and waist/hip circumference were made. The presence of traditional risk factors for cardiovascular disease were assessed in all study participants including blood pressure (BP), and analysis of fasting venous blood which was collected into S‐Monovette tubes (Sarstedt AG and Co.) pre‐prepared with sodium fluoride/ethylenediamine tetra‐acetic acid (EDTA), tri‐potassium‐EDTA (K_3_‐EDTA) and clotting activator/gel and for analysis of glucose, glycosylated haemoglobin triglycerides, and lipid profile respectively.

Participants also had non‐invasive measurement of arterial function by using brachial artery oscillometry to calculate pulse wave velocity (PWV; normal: 7–9.7 m/s; Arteriograph, Tensiomed Ltd)^16^; a predictor of future cardiovascular events and all‐cause mortality.^17^

#### 2.3.3 Assessment of wellbeing and psychosocial functioning

We used the Hospital Anxiety and Depression Scale (HADS) to assesses symptoms of depression and anxiety in the study group.^18^ Subscale symptom scores can be categorised as normal (scores of 0–7), mild (scores of 8–10), moderate (scores of 11–14), and severe (scores of 15–21).^19^ Participants also completed the 36‐Item Short Form Health Survey (SF‐36), which is a patient‐reported tool used to assess physical and mental health across 8 multi‐item health variables describing physical functioning, role limitations due to physical limitations, pain, general perception of health, social functioning, role limitations due to emotional limitations, mental health and energy.^20^ For each variable individual item scores are mapped onto a scale from 0 (worst possible) to 100 (best possible health state).^20^ Additionally, the tool generates two summary measures for physical and mental health — the physical component summary (PCS) and mental component summary (MCS). A 5‐point change in SF‐36 PCS and MCS scales represents a minimal clinically important difference (MCID).^21^

#### 2.3.4 Assessment of functional capacity

Evaluation of functional capacity (Supporting Information S1) comprised participants completing the following: 30‐s sit‐to‐stand,^22^ timed up‐and‐go,^23^ single leg balance test,^24^ and static body‐weight wall‐squat (SBWS).^25^

### 2.4 Statistical analysis

Statistical analysis was performed using IBM SPSS Statistics version 28.0.1.0 software (Armonk). Sample size calculation based on a 5‐point change in DLQI, assuming a standard deviation (SD) of 5.14,^26^ and a 5mmHg change in systolic BP, assuming a SD of 6.32 mmHg^27^ identified target recruitment of 18 participants to detect changes in DLQI and BP, with a power of 0.8, an alpha level set at 0.05, and allow for a 20% dropout rate. Normality of data was tested using the Shapiro–Wilk test. Comparisons between pre‐, post‐intervention and end of study timepoints were made using one‐way ANOVA with repeated‐measures testing, or with the Friedman test followed by post‐hoc Wilcoxon signed ranks testing for non‐normally distributed data. Missing data was imputed using the last observation carried forward method. Descriptive statistics were presented as mean/standard deviation and median/inter‐quartile range (IQR) for normal and non‐normally distributed variables respectively. Percentages were used for categorical variables. Significance was set at *p* < 0.05.

## 3 RESULTS

The study cohort comprised 8 men and 8 women, with a mean age of 39.2 ± 10.1 years old, median BMI of 27.1 (IQR 24.8–32.0), and of whom 18.8% had PsA. Detailed demographic and descriptive data of the study participants are outlined in Table 1, who had a range of psoriasis severities (PASI ≤12.3). In total, 56.3% of participants (*n* = 9) completed all 20 sessions comprising the PA intervention, with only 18.75% (*n* = 3) unable to attend 1 session and 25% (*n* = 4) who did not attend 2 sessions.

**TABLE 1:** Baseline characteristics of the patients with psoriasis.

Characteristic	All participants (*n* = 16)	Men (*n* = 8)	Women (*n* = 8)
Age, years	39.2 ± 10.1	38.9 ± 12.1	39.5 ± 8.5
Body mass index, kg/m^2^	27.1 (24.8–32.0)	26.1 (24.1–28.9)	30.8 (25.1–32.0)
Assessments of psoriasis			
PASI, (0–72)	4.0 (1.7–5.5)	3.8 (1.7–5.9)	4.1 (2.3–5.5)
PGA, (0–6)	3.0 (1.8–4.0)	3.5 (1.8–4.3)	2.5 (1.8–4.0)
DLQI, (0–30)	6.0 (3.0–9.0)	4.0 (3.0–12.8)	7.5 (4.0–8.0)
Psoriasis history			
Age of onset, years	19.6 ± 8.1	21.9 ± 5.7	17.3 ± 9.7
Disease duration, years	19.6 ± 11.8	17.1 ± 10.7	22.1 ± 13.0
Family history of psoriasis, *n* (%)	8 (50.0%)	4 (50.0%)	4 (50.0%)
Nail involvement, *n* (%)	7 (43.8%)	5 (62.5%)	2 (25.0%)
Current psoriasis treatment, *n* (%)			
Topical	14 (87.5%)	7 (87.5%)	7 (87.5%)
Phototherapy	0 (0.0%)	0 (0.0%)	0 (0.0%)
Systemic	1 (6.3%)	0 (0.0%)	1 (12.5%)
Biological	2 (12.5%)	2 (25.0%)	0 (0.0%)
Previous psoriasis treatment, *n* (%)			
Topical	15 (93.8%)	7 (87.5%)	8 (100.0%)
Phototherapy	3 (18.8%)	2 (25.0%)	1 (12.5%)
Systemic	3 (18.8%)	2 (25.0%)	1 (12.5%)
Biological	2 (12.5%)	2 (25.0%)	0 (0.0%)
Past medical history, *n* (%)			
Psoriatic arthritis	3 (18.8%)	1 (12.5%)	2 (25.0%)
Cardiovascular disease	1 (6.3%)	1 (12.5%)	0 (0.0%)
Chronic kidney disease	0 (0.0%)	0 (0.0%)	0 (0.0%)
Hypertension	5 (31.3%)	3 (37.5%)	2 (25.0%)
Angina	2 (12.5%)	2 (25.0%)	0 (0.0%)
Rheumatoid arthritis	0 (0.0%)	0 (0.0%)	0 (0.0%)
Diabetes mellitus	1 (6.3%)	1 (12.5%)	0 (0.0%)
Depression	3 (18.8%)	2 (25.0%)	1 (12.5%)
Asthma	3 (18.8%)	1 (12.5%)	2 (25.0%)

*Note*: The data are presented as mean/standard deviation and median/inter‐quartile range for normal and non‐normally distributed variables. Percentages are used for categorical variables.

Abbreviations: DLQI, Dermatology Life Quality Index; *n*, number of participants; PASI, Psoriasis Area and Severity Index; PGA, Physician Global Assessment.

### 3.1 Psoriasis and PsA disease activity was significantly reduced at week‐10 and ‐20

**FIGURE 2:**
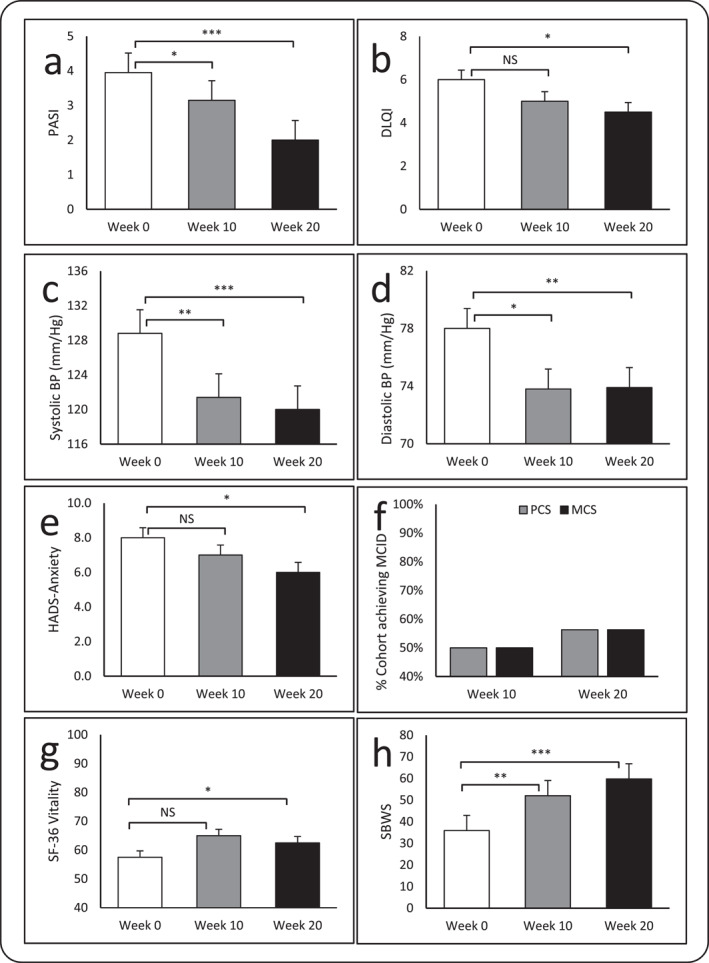
The effects of our physical activity intervention on the following health‐related parameters: (a) PASI at week‐10 (*p* = 0.01) and ‐20 (*p* = <0.001), (b) DLQI at week‐10 (*p* = 0.054) and ‐20 (*p* = 0.04), (c) Systolic blood pressure (BP) at week‐10 (*p* = 0.002) and ‐20 (*p* = 0.001), (d) Diastolic BP at week‐10 (*p* = 0.03) and ‐20 (*p* = 0.008), (e) HADS‐Anxiety at week‐10 (*p* = 0.833) and ‐20 (*p* = 0.04), (f) SF‐36, physical component summary (PCS) and MCS MCID response at week‐10 and ‐20, (g) SF‐36 Energy at week‐10 (*p* = 0.277) and ‐20 (*p* = 0.04), and (h) SBWS at week‐10 (*p* = 0.005) and ‐20 (*p* = <0.001). Data are presented as mean/standard deviation, and median/inter quartile range. All assessments were completed by 16 participants, except for step count which includes 14 participants. BP, blood pressure; DLQI, Dermatology Life Quality Index; HADS, Hospital Anxiety and Depression Scale; MCID, minimal clinically important difference; MCS, mental component summary; PASI, Psoriasis Area and Severity Index; PCS, physical component summary; SBWS, static bodyweight wall‐squat; SF‐36, 36‐Item Short Form Health Survey.

We observed significantly reduced PASI at week‐10 (*p* = 0.01) and ‐20 (*p* = 0.001) compared to baseline (Figure 2a), with 18.8% and 50% of participants achieving PASI‐50 at week‐10 and ‐20, respectively. Significantly reduced PGA was documented at week‐10 (*p* = 0.01) and ‐20 (*p* = 0.002; Table 2). Indeed, DLQI was significantly reduced at week‐20 (*p* = 0.04), compared to baseline (Table 2; Figure 2b). Indeed, a 5‐point reduction in DLQI (or DLQI of 1 or 0, in those with baseline DLQI of ≥3), was achieved in 35.7% of participants at week‐20. We observed a reduction in indicators of PsA activity with PsAID‐12 reduced at week‐10 (median reduction −6.1%) and week‐20 (median reduction −56.1%), and RAPID3 reduced at week‐10 (median reduction −36.4%) and week‐20 (median reduction −63.6%), although this did not reach statistical significance (Table 2).

**TABLE 2:** Assessments of psoriasis, psoriatic arthritis, and cardiometabolic health outcomes, at baseline (week 0), following of the physical activity programme (week 10), and upon completion of the study (week 20).

Characteristic	Baseline (week 0)	Completion of PA programme (week 10)	Completion of the study (week 20)	*p* Value	
				0–10	0–20
Psoriasis					
PASI, (0–72)	4.0 (1.7–5.5)	3.2 (0.7–4.4)	1.9 (0.4–3.2)	0.01	<0.001
PGA, (0–6)	3.0 (1.8–4.0)	2.0 (1.0–3.3)	1.0 (1.0–3.0)	0.01	0.002
DLQI, (0–30)	6.0 (3.0–9.0)	5.0 (4.0–7.5)	4.5 (3.0–8.0)	0.054	0.04
PsAID‐12, (0–10)	6.6 (5.4–7.5)	6.2 (3.1–7.5)	2.9 (1.5–3.6)	0.593	0.102
RAPID3, (0–30)	11.0 (9.5–11.5)	7.0 (4.0–7.0)	4.0 (2.0–7.5)	0.109	0.180
Cardiometabolic status					
Body mass index, kg/m^2^	27.1 (24.8–32.0)	26.7 (25.1–31.4)	27.1 (25.3–31.2)	0.930	0.930
Body weight, kg	79.9 (73.4–87.4)	81.0 (73.7–88.3)	82.0 (73.5–86.2)	0.930	0.930
Waist to hip ratio, index	0.86 (0.83–0.94)	0.90 (0.82–0.94)	0.86 (0.81–0.92)	0.377	0.133
Waist circumference, cm	96.0 (88.0–102.0)	90.5 (85.1–101.0)	89.7 (85.0–94.9)	0.04	0.05
Hip circumference, cm	107.8 (102.2–112.0)	104.1 (100.5–107.5)	104.8 (99.9–109.2)	<0.001	<0.001
Pulse wave velocity, m/s	6.8 (6.5–8.1)	7.2 (6.6–8.0)	6.6 (6.3–7.6)	0.293	0.02
Systolic BP, mmHg	128.8 ± 13.9	121.4 ± 13.8	120.0 ± 13.3	0.002	0.001
Diastolic BP, mmHg	78.0 ± 9.4	73.8 ± 11.0	73.9 ± 9.3	0.03	0.008
HbA1c, mmol/mol	38.6 ± 5.0	39.6 ± 5.7	39.1 ± 5.2	0.063	0.185
Glucose, mmol/L	5.1 (4.9–5.6)	4.9 (4.8–5.1)	5.1 (4.7–5.2)	0.04	0.536
Cholesterol, mmol/L	4.8 ± 0.8	4.8 ± 0.8	4.7 ± 0.8	0.755	0.232
HDL‐cholesterol, mmol/L	1.2 (1.0–1.7)	1.2 (1.1–1.8)	1.3 (1.1–1.5)	0.596	0.596
LDL‐cholesterol, mmol/L	2.7 ± 0.6	2.9 ± 0.7	2.7 ± 0.8	0.349	0.945
Triglycerides, mmol/L	1.4 (1.0–2.3)	1.0 (1.0–1.5)	1.2 (0.9–1.4)	0.216	0.724
Non‐HDL cholesterol, mmol/L	3.5 ± 0.8	3.4 ± 0.8	3.2 ± 1.1	0.662	0.145
Psychological health					
HADS, (0–21)					
HADS‐depression	3.5 (2.0–6.8)	2.0 (1.0–7.5)	2.0 (1.8–3.3)	0.331	0.157
HADS‐anxiety	8.0 (4.8–10.3)	7.0 (5.0–11.0)	6.0 (3.8–9.0)	0.833	0.04
SF‐36, (0–100)					
SF‐36 physical functioning	92.5 (80.0–100)	95.0 (90.0–100)	97.5 (90.0–100)	0.426	0.112
SF‐36 role limitations due to physical limitations	100 (68.0–100)	100 (100–100)	100 (93.8–100)	1.000	1.000
SF‐36 pain	90.0 (57.5–100)	85.0 (69.5–100)	77.5 (68.0–90.0)	0.157	0.480
SF‐36 general health perceptions	60.0 (45.0–71.3)	65.0 (56.3–75.0)	70.5 (60.0–80.0)	0.860	0.052
SF‐36 social functioning	66.7 (0–100)	100 (75.0–100)	100 (25.0–100)	0.377	0.659
SF‐36 role limitations due to emotional limitations	75.0 (53.1–100)	87.5 (71.9–100)	93.8 (62.9–100)	0.289	0.426
SF‐36 mental health	72.0 (43.0–77.0)	72.0 (55.0–84.0)	76.0 (60.0–85.0)	0.190	0.01
SF‐36 energy	57.5 (43.8–70.0)	65.0 (45.0–75.0)	62.5 (47.5–81.3)	0.277	0.04
SF‐36 physical component summary	77.8 (64.8–91.3)	81.6 (69.8–91.3)	83.1 (81.1–89.3)	0.480	0.01
SF‐36 mental component summary	70.6 (47.9–77.1)	79.0 (54.2–87.7)	83.0 (46.9–89.7)	0.289	0.02
Functional capacity					
30‐s sit‐to‐stand, number of chair stands	12.4 ± 2.2	14.0 ± 2.6	13.8 ± 3.3	0.001	0.01
Timed up and go, s	7.31 (6.76–8.14)	6.41 (5.96–7.22)	6.36 (5.55–7.35)	0.001	0.004
Single leg balance, s	54.26 (41.52–60.00)	60.00 (42.93–60.00)	60.00 (55.40–60.00)	0.678	0.008
Static body‐weight wall‐squat, s	36.11 (24.39–46.17)	49.22 (42.47–70.14)	60.39 (46.34–73.55)	0.005	<0.001
Step count, steps	5417 ± 2645	8199 ± 3289	6020 ± 3348	0.001	0.4

*Note*: The data are presented as mean/standard deviation and median/inter‐quartile range for normal and non‐normally distributed variables. *N* = 16 for all variables, except for PsAID‐12 and RAPID3 assessments which had *n* = 3, and step count which had *n* = 14.

Abbreviations: BP, blood pressure; DLQI, Dermatology Life Quality Index; HADS, Hospital Anxiety and Depression Scale; HbA1c, Glycated haemoglobin; HDL, high‐density lipoprotein; LDL, Low‐density lipoprotein; PA, Physical activity; PASI, Psoriasis Area and Severity Index; PGA, Physician Global Assessment; PsA, psoriatic arthritis; PsAID‐12, Psoriatic Arthritis Impact of Disease 12‐item questionnaire; RAPID3, Routine Assessment of Patient Index Data 3; s, seconds; SF‐36, 36‐Item Short Form Health Survey.

### 3.2 Cardiometabolic disease status was significantly improved at week‐10 and ‐20

Body weight and BMI was unchanged throughout the study (Table 2). Significant reductions in hip circumference at week‐10 (median reduction 3.7 cm; *p* = <0.001) and week‐20 (median reduction 3.0 cm; *p* = <0.001) were observed (Table 2). Waist circumference was significantly reduced at week‐10 (median reduction 5.5 cm; *p* = 0.04), which remained reduced at week‐20 (median reduction 6.3 cm; *p* = 0.05), compared to baseline, but waist‐to‐hip ratio did not achieve statistical significance (Table 2). Blood pressure was significantly reduced at week‐10 (systolic: −7.4 mmHg, *p* = 0.002; diastolic: −4.2 mmHg, *p* = 0.03) and week‐20 (systolic: −8.8 mmHg, *p* = 0.001; diastolic: 4.1 mmHg, *p* = 0.008) compared to baseline (Figure 2c and d).

Fasting glucose was significantly reduced at week‐10 (*p* = 0.04), compared to baseline. Fasting lipids were not significantly reduced at week‐10 and week‐20, compared to baseline (Table 2). Fasting triglyceride levels were reduced at week‐10 and ‐20, compared to baseline, although this was not significant. All other components of the fasting lipid profile were unchanged throughout the study, although sub‐group analysis demonstrated a significant reduction in non‐HDL cholesterol amongst women at week‐20 (−0.4 mmol/L, *p* = 0.04; *n* = 8), compared to baseline. We also documented a significant reduction in PWV at week‐20 (median reduction 0.2 m/s, *p* = 0.02) compared to baseline suggesting improvement in cardiovascular health (Table 2).

### 3.3 Enhanced psychological health following completion of our study

We observed high prevalence of anxiety and depression at baseline with 56.3% of patients having anxiety scores (HADS‐A) of ≥8 (25% having HADS‐A ≥ 11) and, 25.0% of patients had depression scores (HADS‐D) of ≥8 (12.5% had HADS‐D ≥ 11). Indeed, 18.8% of all patients had a self‐reported history of depression and 6.3% used antidepressant prescription medications.

However, patients had significantly improved HADS‐A at week‐20 (median: 6.0, IQR: 3.8–9.0, *p* = 0.04) compared with baseline (median: 8.0, IQR: 4.8–10.3; Table 2; Figure 2e). In addition, a significantly greater proportion of patients with anxiety at baseline (HADS‐A ≥8: 56.3%) changed their clinical status by achieving a HADS‐A score of <8 at week‐20 (HADS‐A ≥ 8: 25.0%, *p* = 0.006). Although, HADS‐D also improved by week‐20 (median: 2.0, IQR: 1.8–3.3, *p* = 0.18) in comparison to baseline (median: 3.5, IQR: 2.0–6.8) this was not significant. Those with depression at baseline (HADS‐D ≥ 8: 25.0%) also demonstrated improved clinical status with a HADS‐D score of <8 at week‐20 (HADS‐D ≥ 8: 18.8%, *p* = 0.62), but this was not significant.

By week‐20, improvements in SF‐36 PCS (median: 83.1, IQR: 81.1–89.3, *p* = 0.01), and MCS (median: 83.0, IQR: 46.9–98.7, *p* = 0.02), in comparison to baseline (PCS – median: 77.8, IQR: 64.8–91.3; MCS – median: 70.6, IQR: 47.9–77.1; Table 2) were observed. Significant improvements in 2/8 domain scores were also observed at week‐20 for mental health (median 76.0, IQR: 60.0–85.0, *p* = 0.01) and energy (median 62.5, IQR: 47.5–81.3, *p* = 0.04), in comparison to baseline (mental health—median 72.0, IQR: 43.0–77.0; energy—median 57.5, IQR: 43.8–70.0; Table 2; Figure 2g). A MCID response for both PCS and MCS was achieved in 50% patients at Week 10% and 56.3% Week 20 (Figure 2f).

### 3.4 Improved measures of functional capacity at week‐10 and ‐20 endpoints

Patients had significantly improved 30‐s sit‐to‐stand (*p* = 0.001), timed up‐and‐go (*p* = 0.001) and SBWS (*p* = 0.005; Figure 2h) at week‐10, and all measures of functional capacity had improved by week‐20 (*p* < 0.020), compared to baseline (Table 2). Step count was significantly increased at week‐10 (8199 ± 3289; *p* = 0.004), which increased at the week‐20 endpoint but did not achieve statistical significance (6020 ± 3348; *p* = 0.4), compared to baseline (5417 ± 2645; Table 2).

## 4 DISCUSSION

This study is the first to investigate the potential health benefits of increased PA in patients with chronic plaque psoriasis, without simultaneous modification of other lifestyle factors such as diet. We report a significant improvement in psoriasis, as measured by PASI and PGA, with 50% of our cohort achieving a PASI‐50 response at week‐20. We observed a significant improvement in cardiometabolic disease status, psychological health, and functional capacity. We also observed 97% adherence to our PA intervention, which was cost‐effective, required no specialised equipment, training, or previous experience.

It is well established that adherence to exercise regimens within the general population is poor,^28^ which may be further reduced in patients with chronic disease who experience symptoms of pain and fatigue.^29^ Indeed, successful adherence to an exercise programme is generally accepted when participants complete more than two‐thirds of the prescribed sessions.^30^ In this study we observed 97% adherence to our intervention, which was completed by an equal number of men and women including 43.8% of participants who were from minority ethnic backgrounds (individuals from minority ethnic backgrounds comprise 23.6% of the population of Greater Manchester).^31^ Taken together these data suggest our programme was both acceptable to our target population and importantly was accessible to all regardless of gender or background. We believe this was achieved by co‐designing the programme in partnership with patients with psoriasis^11^ and our previous work to understand and address existing barriers to engagement with physical activities.^1^

Our study suggests that increased PA may offer utility in the management of psoriasis and psoriasis comorbidities. Following our intervention, 50% of patients achieved PASI‐50, compared to baseline. In comparison, 39.9% of patients with psoriasis, with a mean age of 43.1 ± 12.9 years old (*n* = 163), mean body weight of 82.0 kg ± 18.6, and of whom 17.2% (*n* = 28) had PsA, receiving methotrexate treatment at a dose of 5–25 mg/week achieved a PASI‐50 after 24‐week^32^ Our study was not adequately powered to detect changes in PsA. However, by week‐20, those with PsA achieved a median reduction in PsAID‐12 and RAPID3 of 56.1% and 63.6%, compared to baseline assessment, which was similar to reporting from other adequately powered studies.^33^ Cardiometabolic disease status improved as evidenced by significant changes in waist and hip circumference, BP, fasting glucose, and PWV. This is in keeping with other walking interventions reporting meaningful reductions in BP amongst populations with rheumatoid arthritis.^34^ We also observed a significantly reduced median PWV of 0.2 m/s at week‐20, compared to baseline, although it has been reported that a 1 m/s reduction constitutes a clinically meaningful change.^17^ Compared to baseline, significant reductions in hip circumference at week‐10 and ‐20 were observed. However, body weight and BMI remained unchanged; similar to studies in patients with rheumatoid arthritis and Type 2 diabetes mellitus.^34^, ^35^ We also observed a median reduction in triglycerides of 0.4 mmol/L at week‐10, compared to baseline, which we anticipated given our previous report of significantly reduced triglycerides in healthy older adults following engagement with a light‐intensity PA protocol.^36^ Compared to baseline, patients had significantly increased step counts at week‐10, which then in the absence of Sports and Exercise Scientist‐led activity, reduced from week‐10 to −20, whilst remaining significantly greater than baseline levels. This is important for two reasons: (i) 7857 steps/day are needed to meet the health‐promoting levels of PA advocated in current guidelines and (ii) each 2000 daily step increment above baseline is associated with a 10% lower cardiovascular event rate (following adjustment for change in BMI and other confounding factors).^37^, ^38^ In our study, mean step count increased by 2782 steps from baseline to week‐10, suggesting patients may have had a 10% reduced risk of future cardiovascular events.

Previous studies have demonstrated that increased PA can improve quality of life, cognitive function, and energy.^4^ Indeed, our study shows that increasing PA may enhance psychological health for those with psoriasis. These data are encouraging given psoriasis is associated with significant psychosocial impairment,^39^ and are consistent with other studies using walking interventions in stroke, overweight/obese, and coronary artery disease patient groups.^40^, ^41^, ^42^ However, not all markers of psychological health were significantly different following our intervention. This may be because we limited our walking group sizes to comply with the national COVID‐19 restrictions which may have impeded social interaction.

Our work had limitations. First, self‐selection bias may have influenced the composition of our study group. However, our final psoriasis cohort comprised an equal group of men and women, with a range of psoriatic comorbidities across broad age ranges. Second, our study was designed to have a single experimental arm similar to other studies in patients with chronic inflammatory diseases over the last decade.^34^, ^43^, ^44^ However, our data suggests that PA could constitute a promising therapeutic intervention for psoriasis and therefore further investigation with an appropriately powered randomised controlled trial is recommended. Third, it is possible a 12‐week PA intervention rather than our 10‐week programme is needed to determine whether clinically meaningful changes in PWV and other cardiovascular outcomes can be elicited, or not.^45^ Indeed, future work would also benefit from assessment of body composition, which was outside the scope of the current study, as change in lean‐body mass may account for the reduction in waist and hip circumference observed despite no decrease in BMI. Fourth, it was out with the scope of the study to assess adherence to treatments for psoriasis, which may have influenced the health outcomes reported. However, only patients with stable disease involving no medication/dose changes for 2‐month prior to recruitment were eligible for participation. Finally, national rules on social gatherings due to COVID limited the size of our groups to *n* = 6 throughout the data collection period. However, we mitigated for this by keeping group sizes similar throughout the study.

## 5 CONCLUSION

Increasing PA constitutes a promising therapeutic intervention in the management of psoriasis, which may also facilitate personalised therapy regimens or targeted adjunctive therapy for patients demonstrating incomplete response to standard therapy. Evaluation of our intervention in a clinical trial would help determine clinical utility and establish PA guidelines for patients with psoriasis.

## CONFLICT OF INTEREST STATEMENT

The authors declare no conﬂicts of interest.

## AUTHOR CONTRIBUTIONS

**Rory Sheppard**: Data curation (lead); formal analysis (lead); investigation (lead); methodology (lead); validation (lead); writing – original draft (lead). **Weh K. Gan**: Data curation (supporting); investigation (supporting); methodology (supporting); writing – review & editing (supporting). **Gladys L. Onambele‐Pearson**: Data curation (supporting); formal analysis (supporting); investigation (supporting); methodology (supporting); software (supporting); supervision (supporting); validation (supporting); writing – review & editing (supporting). **Helen S. Young**: Conceptualization (lead); data curation (supporting); formal analysis (supporting); funding acquisition (lead); investigation (supporting); methodology (supporting); project administration (lead); resources (lead); software (supporting); supervision (lead); validation (lead); visualization (lead); writing – original draft (supporting); writing – review & editing (lead).

## ETHICS STATEMENT

The study was approved by the Local Research Ethics Committee (20/NW/0443).

## PATIENT CONSENT

Not applicable.

## Supporting information

Supporting Information S1

## Data Availability

The data underlying this article are available in the article and in its online supplementary material.

## References

[1] Auker L , Cordingley L , Pye SR , Griffiths C , Young H . What are the barriers to physical activity in patients with chronic plaque psoriasis? Br J Dermatol. 2020;183(6):1094–1102. 10.1111/bjd.18979 32107775 PMC7754450

[2] Auker L , Cordingley L , Griffiths CEM , Young HS . Physical activity is important for cardiovascular health and cardiorespiratory fitness in patients with psoriasis. Clin Exp Dermatol. 2022;47(2):289–296. 10.1111/ced.14872 34368977 PMC9291751

[3] Health Survey for England . Health Survey for England ‐:2012. [WWW Document]. 2012.URL https://digital.nhs.uk/data‐and‐information/publications/statistical/health‐survey‐for‐england/health‐survey‐for‐england‐2012 accessed on 1 May 2024.

[4] Garber CE , Blissmer B , Deschenes MR , Franklin BA , Lamonte MJ , Lee IM , et al. American College of Sports Medicine position stand. Quantity and quality of exercise for developing and maintaining cardiorespiratory, musculoskeletal, and neuromotor fitness in apparently healthy adults: guidance for prescribing exercise. Med Sci Sports Exerc. 2011;43(7):1334–1359. 10.1249/mss.0b013e318213fefb 21694556

[5] Reid C , Griffiths CEM . Psoriasis and treatment: past, present and future aspects. Acta Derm Venereol. 2020;100(3):69–79. 10.2340/00015555-3386 PMC9128930 31971601

[6] Lebwohl MG , Barker J , Girolomoni G , Kavanaugh A , Langley RG , et al. Patient perspectives in the management of psoriasis: results from the population‐based multinational assessment of psoriasis and psoriatic arthritis survey. J Am Acad Dermatol. 2014;70(5):871–881. 10.1016/j.jaad.2013.12.018 24576585

[7] Raychaudhuri SP , Gross J . Psoriasis risk factors: role of lifestyle practices. Cutis. 2000;66:348–352. 11107520

[8] Ruegsegger GN , Booth FW . Health benefits of exercise. Cold Spring Harb Perspect Med. 2018;8(7):a029694. 10.1101/cshperspect.a029694 28507196 PMC6027933

[9] Majeed‐Ariss R , McPhee M , McAteer H , Griffiths C , Young H . The top 10 research priorities for psoriasis in the UK: results of a James Lind Alliance psoriasis Priority Setting Partnership. Br J Dermatol. 2019;181(4):871–873. 10.1111/bjd.18209 31162641 PMC6973084

[10] Hailey L , Bundy C , Burstow H , Chandler D , Cowper R , Helliwell P , et al. The top 10 research priorities in psoriatic arthritis: a James Lind alliance priority setting partnership. Rheumatology. 2023;62(8):2716–2723. 10.1093/rheumatology/keac676 36453848 PMC10393438

[11] Sheppard R , Gan WK , Onambele‐Pearson GL , Young HS . Developing an aerobic exercise intervention for patients with psoriasis to support lifestyle behaviour change and improve health outcomes. Clin Exp Dermatol. 2023;48:5–11. 36669177 10.1093/ced/llac008

[12] Fredriksson T , Pettersson U . Severe psoriasis–oral therapy with a new retinoid. Dermatology. 1978;157(4):238–244. 10.1159/000250839 357213

[13] Finlay AY , Khan GK . Dermatology Life Quality Index (DLQI)—a simple practical measure for routine clinical use. Clin Exp Dermatol. 1994;19(3):210–216. 10.1111/j.1365-2230.1994.tb01167.x 8033378

[14] Gossec L , de Wit M , Kiltz U , Braun J , Kalyoncu U , Scrivo R , et al. A patient‐derived and patient‐reported outcome measure for assessing psoriatic arthritis: elaboration and preliminary validation of the Psoriatic Arthritis Impact of Disease (PsAID) questionnaire, a 13‐country EULAR initiative. Ann Rheum Dis. 2014;73(6):1012–1019. 10.1136/annrheumdis-2014-205207 24790067

[15] Pincus T , Swearingen CJ , Bergman M , Yazici Y . RAPID3 (Routine Assessment of Patient Index Data 3), a rheumatoid arthritis index without formal joint counts for routine care: proposed severity categories compared to disease activity score and clinical disease activity index categories. J Rheumatol. 2008;35(11):2136–2147. 10.3899/jrheum.080182 18793006

[16] Ikonomidis I , Makavos G , Lekakis J . Arterial stiffness and coronary artery disease. Curr Opin Cardiol. 2015;30(4):422–431. 10.1097/hco.0000000000000179 26049393

[17] Vlachopoulos C , Aznaouridis K , Stefanadis C . Prediction of cardiovascular events and all‐cause mortality with arterial stiffness: a systematic review and meta‐analysis. J Am Coll Cardiol. 2010;55(13):1318–1327. 10.1016/j.jacc.2009.10.061 20338492

[18] Zigmond AS , Snaith RP . The hospital anxiety and depression scale. Acta Psychiatr Scand. 1983;67(6):361–370. 10.1111/j.1600-0447.1983.tb09716.x 6880820

[19] Stern AF . The hospital anxiety and depression scale. Occup Med (Chic Ill). 2014;64(5):393–394. 10.1093/occmed/kqu024 25005549

[20] Ware JJE . SF‐36 health survey update. Spine. 2000;25(24):3130–3139. 10.1097/00007632-200012150-00008 11124729

[21] Coteur G , Feagan B , Keininger DL , Kosinski M . Evaluation of the meaningfulness of health‐related quality of life improvements as assessed by the SF‐36 and the EQ‐5D VAS in patients with active Crohn’s disease. Aliment Pharmacol Ther. 2009;29(9):1032–1041. 10.1111/j.1365-2036.2009.03966.x 19222413

[22] Jones CJ , Rikli RE . Measuring functional fitness of older adults. J Act aging. 2002;1:24–30.

[23] Herman T , Giladi N , Hausdorff JM . Properties of the ‘timed up and go’test: more than meets the eye. Gerontology. 2011;57(3):203–210. 10.1159/000314963 20484884 PMC3094679

[24] Freeman MAR , Dean MRE , Hanham IWF . The etiology and prevention of functional instability of the foot. J Bone Jt Surg Br. 1965;47(4):678–685. 10.1302/0301-620x.47b4.678 5846767

[25] Biscarini A , Contemori S , Dieni CV , Panichi R . Joint torques and tibiofemoral joint reaction force in the bodyweight “Wall Squat” therapeutic exercise. Appl Sci. 2020;10(9):3019. 10.3390/app10093019

[26] Ersser SJ , Cowdell FC , Nicholls PG , Latter S , Healy E . A pilot randomized controlled trial to examine the feasibility and efficacy of an educational nursing intervention to improve self‐management practices in patients with mild‐moderate psoriasis. J Eur Acad Dermatol Venereol. 2012;26(6):738–745. 10.1111/j.1468-3083.2011.04158.x 21707770

[27] Patil SG , Aithala MR , Das KK . Effect of yoga on arterial stiffness in elderly subjects with increased pulse pressure: a randomized controlled study. Compl Ther Med. 2015;23(4):562–569. 10.1016/j.ctim.2015.06.002 26275649

[28] Collado‐Mateo D , Lavín‐Pérez AM , Peñacoba C , Del Coso J , Leyton‐Román M , Luque‐Casado A , et al. Key factors associated with adherence to physical exercise in patients with chronic diseases and older adults: an umbrella review. Int J Environ Res Publ Health. 2021;18(4):1–24. 10.3390/ijerph18042023 PMC7922504 33669679

[29] Craike M , Gaskin CJ , Courneya KS , Fraser SF , Salmon J , Owen PJ , et al. Predictors of adherence to a 12‐week exercise program among men treated for prostate cancer: ENGAGE study. Cancer Med. 2016;5:787–794. 10.1002/cam4.639 26872005 PMC4864808

[30] Hawley‐Hague H , Horne M , Skelton DA , Todd C . Review of how we should define (and measure) adherence in studies examining older adults’ participation in exercise classes. BMJ Open. 2016;6:e011560. 10.1136/bmjopen-2016-011560 PMC4932302 27338884

[31] Varbes . Demographics of greater manchester [WWW document]. 2021. URL https://www.varbes.com/demographics/greater‐manchester‐demographics. accessed on 27 June 2023.

[32] Reich K , Langley RG , Papp KA , Ortonne JP , Unnebrink K , Kaul M , et al. A 52‐week trial comparing briakinumab with methotrexate in patients with psoriasis. N Engl J Med. 2011;365(17):1586–1596. 10.1056/nejmoa1010858 22029980

[33] Thomsen RS , Nilsen TIL , Haugeberg G , Bye A , Kavanaugh A , Hoff M . Impact of high‐intensity interval training on disease activity and disease in patients with psoriatic arthritis: a randomized controlled trial. Arthritis Care Res. 2019;71(4):530–537. 10.1002/acr.23614 29882634

[34] Bartlett DB , Willis LH , Slentz CA , Hoselton A , Kelly L , Huebner JL , et al. Ten weeks of high‐intensity interval walk training is associated with reduced disease activity and improved innate immune function in older adults with rheumatoid arthritis: a pilot study. Arthritis Res Ther. 2018;20:1–15. 10.1186/s13075-018-1624-x 29898765 PMC6001166

[35] Gram B , Christensen R , Christiansen C , Gram J . Effects of nordic walking and exercise in type 2 diabetes mellitus: a randomized controlled trial. Clin J Sport Med. 2010;20:355–361. 20818193 10.1227/NEU.0b013e3181e56e0a

[36] Grant D , Tomlinson D , Tsintzas K , Kolić P , Onambele‐Pearson GL . The effects of displacing sedentary behavior with two distinct patterns of light activity on health outcomes in older adults (implications for COVID‐19 quarantine). Front Physiol. 2020;11. 10.3389/fphys.2020.574595 PMC7793876 33424618

[37] Cao Z.‐B , Oh T , Miyatake N , Tsushita K , Higuchi M , Tabata I . Steps per day required for meeting physical activity guidelines in Japanese adults. J Phys Activ Health. 2014;11(7):1367–1372. 10.1123/jpah.2012-0333 24366861

[38] Yates T , Haffner SM , Schulte PJ , Thomas L , Huffman KM , Bales CW , et al. Association between change in daily ambulatory activity and cardiovascular events in people with impaired glucose tolerance (NAVIGATOR trial): a cohort analysis. Lancet. 2014;383(9922):1059–1066. 10.1016/s0140-6736(13)62061-9 24361242

[39] Fortune DG , Main CJ , O’Sullivan TM , Griffiths CEM . Assessing illness‐related stress in psoriasis: the psychometric properties of the Psoriasis Life Stress Inventory. J Psychosom Res. 1997;42(5):467–475. 10.1016/s0022-3999(97)00036-6 9194019

[40] Aguiar LT , Nadeau S , Britto RR , Teixeira‐Salmela LF , Martins JC , Samora GAR , et al. Effects of aerobic training on physical activity in people with stroke: a randomized controlled trial. NeuroRehabilitation. 2020;46(3):391–401. 10.3233/nre-193013 32250336

[41] Vancini RL , Rayes ABR , Lira CABde , Sarro KJ , Andrade MS . Pilates and aerobic training improve levels of depression, anxiety and quality of life in overweight and obese individuals. Arq Neuropsiquiatr. 2017;75(12):850–857. 10.1590/0004-282x20170149 29236887

[42] Reed JL , Terada T , Cotie LM , Tulloch HE , Leenen FH , Mistura M , et al. The effects of high‐intensity interval training, Nordic walking and moderate‐to‐vigorous intensity continuous training on functional capacity, depression and quality of life in patients with coronary artery disease enrolled in cardiac rehabilitation: a ra. Prog Cardiovasc Dis. 2022;70:73–83. 10.1016/j.pcad.2021.07.002 34245777

[43] Farinha JB , Steckling FM , Stefanello ST , Cardoso MS , Nunes LS , Barcelos RP , et al. Response of oxidative stress and inflammatory biomarkers to a 12‐week aerobic exercise training in women with metabolic syndrome. Sport Med ‐ Open. 2015;1:1–10. 10.1186/s40798-015-0011-2 PMC5005613 26284160

[44] Keech A , Holgate K , Fildes J , Indraratna P , Cummins L , Lewis C , et al. High‐intensity interval training for patients with coronary artery disease: finding the optimal balance. Int J Cardiol. 2019;298:8–14. 10.1016/j.ijcard.2019.09.060 31648826

[45] Brozic AP , Marzolini S , Goodman JM . Effects of an adapted cardiac rehabilitation programme on arterial stiffness in patients with type 2 diabetes without cardiac disease diagnosis. Diabetes Vasc Dis Res. 2017;14:104–112. 10.1177/1479164116679078 28093924

