# Supplementary material for: Increased physical activity promotes skin clearance, improves cardiovascular and psychological health, and increases functional capacity in patients with psoriasis
Source: Skin Health Dis. 2024 Aug 9;4(5):e426. doi: 10.1002/ski2.426 (PMC11442072; doi:10.1002/ski2.426)
Supplement: Supplementary file 1 — Supporting Information S1 [file SKI2-4-e426-s001.doc]

**Supporting information**

*Functional capacity assessment protocol*

All exercise assessments were performed in a clinic room using the following equipment: a chair without armrests, stopwatch, tape measure and cone (to mark the turnaround point).

Participants completed each assessment three times, and the final attempt was recorded.

The Timed Up-and-Go (TUG) is a functional test to assess mobility, lower extremity function and falls risk in human subjects.^22^ This assessment measured how quickly an individual could stand from a seated position, walk three metres (measured by tape measure and marked with a cone), turn around, and return to their original seated position without running.

The 30-second Sit-To-Stand (30STS) assesses physical function,^45^ and is used as a measure of lower-extremity strength.^46^ It provides an indication of individual ability to perform common tasks within a community setting such as walking, stair climbing, changing from a seated position to leave a chair/car.^21^ This test measured how many complete chair stands could be completed, starting from a seated position, within 30-seconds.

The static Single Leg Balance (SLB) test is a simple assessment of postural control.^47^ This assessment measured how long balance could be maintained whilst standing on one-leg for up to 60-seconds. Participants had their eyes open, held their arms at the hips, and maintained a 90-degree knee angle with the non-weight bearing leg. The test and stopwatch was stopped if patients deviated from this position. The stopped time was recorded as the score. Participants stood on the same weight-bearing leg throughout the study.

The Static Body-Weight Wall-Squat (SBWS), also referred to as ‘Samson chair’, tests lower-extremity strength and muscle endurance.^48^ The assessment measured how long an individual could hold a static squatting position against a wall for up to 2-minutes, whilst maintaining a 90-degree knee angle and resting the arms on the legs. The test and stopwatch was stopped if participants deviated from this position. The stopped time was recorded as the score.
